# Text Messaging-Based Interventions for Smoking Cessation: A Systematic Review and Meta-Analysis

**DOI:** 10.2196/mhealth.5436

**Published:** 2016-05-20

**Authors:** Lori A. J Scott-Sheldon, Ryan Lantini, Ernestine G Jennings, Herpreet Thind, Rochelle K Rosen, Elena Salmoirago-Blotcher, Beth C Bock

**Affiliations:** ^1^ Centers for Behavioral and Preventive Medicine The Miriam Hospital Providence, RI United States; ^2^ Department of Psychiatry and Human Behavior Alpert School of Medicine Brown University Providence, RI United States; ^3^ Department of Behavioral and Social Sciences Brown School of Public Health Providence, RI United States; ^4^ Department of Medicine Alpert School of Medicine Brown University Providence, RI United States; ^5^ Department of Epidemiology Brown School of Public Health Providence, RI United States

**Keywords:** text messaging, smoking cessation, intervention, cigarette smoking, meta-analysis

## Abstract

**Background:**

Tobacco use is one of the leading preventable global health problems producing nearly 6 million smoking-related deaths per year. Interventions delivered via text messaging (short message service, SMS) may increase access to educational and support services that promote smoking cessation across diverse populations.

**Objective:**

The purpose of this meta-analysis is to (1) evaluate the efficacy of text messaging interventions on smoking outcomes, (2) determine the robustness of the evidence, and (3) identify moderators of intervention efficacy.

**Methods:**

Electronic bibliographic databases were searched for records with relevant key terms. Studies were included if they used a randomized controlled trial (RCT) to examine a text messaging intervention focusing on smoking cessation. Raters coded sample and design characteristics, and intervention content. Summary effect sizes, using random-effects models, were calculated and potential moderators were examined.

**Results:**

The meta-analysis included 20 manuscripts with 22 interventions (N=15,593; 8128 (54%) women; mean age=29) from 10 countries. Smokers who received a text messaging intervention were more likely to abstain from smoking relative to controls across a number of measures of smoking abstinence including 7-day point prevalence (odds ratio (OR)=1.38, 95% confidence interval (CI)=1.22, 1.55, k=16) and continuous abstinence (OR=1.63, 95% CI=1.19, 2.24, k=7). Text messaging interventions were also more successful in reducing cigarette consumption relative to controls (d_+_=0.14, 95% CI=0.05, 0.23, k=9). The effect size estimates were biased when participants who were lost to follow-up were excluded from the analyses. Cumulative meta-analysis using the 18 studies (k=19) measuring abstinence revealed that the benefits of using text message interventions were established only after only five RCTs (k=5) involving 8383 smokers (OR=1.39, 95% CI=1.15, 1.67, *P*<.001). The inclusion of the subsequent 13 RCTs (k=14) with 6870 smokers did not change the established efficacy of text message interventions for smoking abstinence (OR=1.37, 95% CI=1.25, 1.51, *P*<.001). Smoking abstinence rates were stronger when text messaging interventions (1) were conducted in Asia, North America, or Europe, (2) sampled fewer women, and (3) recruited participants via the Internet.

**Conclusions:**

The evidence for the efficacy of text messaging interventions to reduce smoking behavior is well-established. Using text messaging to support quitting behavior, and ultimately long-term smoking abstinence, should be a public health priority.

## Introduction

Tobacco use is a major preventable public health problem resulting in nearly 6 million deaths from direct tobacco use and second-hand exposure per year [1]. The global economic cost associated with tobacco use is estimated to be over US$1 trillion annually.[2] In the United States alone, tobacco use causes more than 480,000 deaths each year and costs nearly US$300 billion in health care and productivity losses annually [3,4]. The global burden of tobacco use could be reduced if all smokers had access to smoking cessation programs.

The life expectancy of a smoker is shortened by approximately one decade compared with those who have never smoked; however, smokers who quit before the age of 40 can reduce their risk of smoking-related death by 90% [5]. An estimated 90% of smokers attempt to quit (unsuccessfully) without assistance, even though effective evidence-based behavioral smoking treatments are available [6,7]. Numerous barriers exist to accessing traditional in-person treatments including costs, time commitments, and other logistics such as travel and appointment scheduling [8]. New smoking cessation intervention delivery systems that have the capacity to reach smokers effectively and efficiently are urgently needed [9]. Mobile technology offers an innovative way to reach smokers worldwide.

Mobile broadband reaches nearly one-half of the world’s population, and mobile phone text messaging (short message service, SMS) interventions are used by approximately 75% of adults [10]. Thus, text messaging holds great promise as a tool for delivering behavioral interventions that have the ability to reach the vast majority of the population. Interventions delivered through text messaging have been shown to be cost effective [11], and eliminate many barriers to accessing traditional treatments. Text message interventions also offer a variety of advantages. For example, users can also access text messaging services whenever a need exists, and these interventions can be provided to individuals within their own environment and delivered in real-time. The content and timing of messages can be tailored to the individual, enabling the provision of adapted advice and support from evidence-based interventions for smoking cessation, for example, to meet the unique needs of each patient. For these reasons, the design, development, and evaluation of text message-delivered interventions for health promotion, disease prevention, and disease management has greatly increased over the past decade [12].

The use of mHealth is a rapidly expanding area of research and practice [13-18]. Prior reviews of the literature have largely focused on mHealth technologies including text messaging and mobile phone apps, for health promotion, disease prevention, or disease management more broadly; with smoking cessation being one of a number of outcome behaviors [13,18-22]. Three meta-analytic reviews have specifically examined the efficacy of text messaging for smoking cessation [23-25]. Whittaker et al [23] included four randomized controlled trials (RCTs) that involved text messaging [26-29]. Two of the four interventions exclusively used text messages and showed a significant increase in short-term (≤6 weeks) smoking cessation rates relative to controls (risk ratio (RR)=2.18, 95% confidence interval (CI) 1.80-2.65). The other two interventions used a combination of both text messaging and Internet components and showed significant increases in long-term (≤52 weeks) smoking cessation rates (RR=2.03, 95% CI 1.40-2.94). In an updated meta-analysis of mobile phone-delivered interventions (predominately text messaging interventions), Whittaker et al [24] included five RCTs (2 of these studies were included in their previous review) assessing smoking cessation outcomes at longer assessment intervals (ie, ≥6 months) [28-32]. They found that mobile phone interventions resulted in greater smoking cessation rates relative to controls (RR=1.71, 95% CI 1.47-1.99). Most recently, Spohr et al [25] evaluated 13 RCTs assessing text messaging for smoking cessation. Consistent with prior reviews, Spohr et al [25] found text messaging interventions to be more successful at increasing smoking cessation rates relative to control conditions (OR=1.35, 95% CI=1.23, 1.48). Spohr et al [25] also assessed potential moderators of smoking cessation (eg, follow-up length, message frequency), but none of these intervention features moderated the effect of text messaging on smoking cessation. Thus, text messaging interventions have proven to be successful at increasing smoking cessation, but likely due to the small number of studies evaluated, meta-analyses conducted to date have likely been underpowered to detect moderators of text messaging [33]. Thus, important factors (eg, number of text messages) that may strengthen or weaken the efficacy of text messaging interventions remains unknown.

The purpose of this systematic review and meta-analysis is to evaluate the current evidence for text messaging smoking interventions. Our review updates and extends the scope of the prior meta-analytic reviews in five ways. First, we update the prior meta-analytic reviews by including seven RCTs that were omitted from prior reviews. Second, because excluding participants who are lost to follow-up from the analyses may bias effect size (ES) estimates (cf., [34]), we examine differences in the overall ESs based on an intent-to-treat approach and a complete case analysis. Third, we used cumulative random-effects meta-analytic approaches to assess the accumulated evidence for text messaging interventions. Fourth, we assess a broader range of smoking outcomes (eg, number of cigarettes used, nicotine dependence), in addition to smoking abstinence. Finally, we identify the extent to which sample characteristics (eg, gender, age, or geographic region) as well as intervention features (eg, number of text messages sent) moderate the efficacy of text messaging interventions on smoking outcomes.

## Methods

### Overview

This systematic review and meta-analysis was conducted in accordance with the Preferred Reporting Items for Systematic Reviews and Meta-Analyses (PRISMA) [35]. The PRISMA checklist can be found in Multimedia Appendix 1.

### Eligibility Criteria

Studies were included if they (1) examined an individual-level text messaging intervention to promote smoking cessation, (2) used a RCT design, (3) assessed smoking outcomes (eg, abstinence, quit attempt), (4) provided sufficient statistical information to calculate ESs, and (5) were available (including electronic publications and dissertations) by December 31, 2014. Studies that examined text messaging interventions with the goal of maintaining (vs initiating) smoking abstinence among recently quit smokers were excluded (eg, Snuggs, McRobbie [36]).

### Information Sources and Search Strategy

Multiple electronic reference databases (*PubMed* (1946+), *PsycINFO* (1872+), *ProQuest Dissertations and Theses Full Text* (1973+)*, CINAHL* (1981+)*, Global Health* (1973+), *The Cochrane Library* (1992+), *Communication & Mass Media Complete* (1915+)*,* and *EMBASE* (1947+)) were searched using a Boolean search strategy: (tobacco OR smok*) AND (text messag*) OR (cellular phones) OR (cell AND phone) OR (mobile) OR (mobile devices) OR ("short message service") or ("multimedia messaging service") AND (interven* or prevent*). Because many electronic databases have specific search methods (eg, Medical Subject Heading (MeSH) terms used in *PubMED* are not used in other databases such as *PsycINFO*), our basic search strategy was modified to accommodate the specific search parameters for each electronic database. No language restrictions were applied. The electronic reference database searches were initially conducted in April 2014 and updated in January 2015 to ensure that we retrieved all available studies through December 31, 2014. Reference sections of relevant manuscripts (including published reviews obtained through the electronic reference database searches) were also reviewed. Finally, we searched the tables of contents of relevant journals (*Journal of Medical Internet Research*; *Telemedicine & eHealth*) for relevant papers.

### Study Selection

Study titles and abstracts were initially screened for possible inclusion. Full-text manuscripts of potentially relevant records and references from relevant manuscripts were retrieved and reviewed for final inclusion. Studies that fulfilled the inclusion criteria (see Eligibility Criteria) were included. When authors reported details, ancillary information (eg, results from a pilot study), and/or study information across multiple manuscripts, those manuscripts were linked in the database and represented as a single unit and the manuscript reporting the most complete study sample was selected as the primary manuscript.

### Coding and Reliability

Two of three independent coders (LAJSS, RCL, or EGJ) extracted study information (eg, publication year), sample characteristics (eg, gender, ethnicity), design specifics (eg, recruitment method), intervention procedures (eg, number and frequency of text messages), and components (eg, personalized feedback, goal setting) from each study. Methodological quality was assessed using 14 items adapted from validated measures [37,38]. Interrater reliability was assessed for all study, sample, and methodological variables. For the categorical variables, raters agreed on a mean of 79% of the judgments (mean Cohen’s κ =.60). Reliability for the continuous variables yielded an average intraclass correlation coefficient (*ρ*) of 0.89 across categories (median=0.99). Disagreements between pairs of coders were resolved through discussion.

### Study Outcomes

Study outcomes included dichotomous (eg, frequencies) and continuous (eg, means) assessments of smoking cessation including abstinence, quit attempts, and cigarette use. Smoking abstinence was assessed using a number of methods including point prevalence abstinence (ie, abstinence from a specific time-point to follow-up assessment), continuous abstinence (ie, abstinence from quit date to follow-up assessment), prolonged or sustained abstinence (ie, sustained abstinence between two assessments), and repeated point prevalence abstinence (ie, abstinence from at least two specific time-points to follow-up assessments; see Hughes et al [39] for details regarding smoking abstinence definitions). Other measures included making a quit attempt and the quantity of cigarettes smoked per day or week. Finally, we assessed nicotine dependence, which was measured using validated measures (eg, Heaviness of Smoking Index [40]).

### Effect Size Calculations

ESs were calculated for each study by two of the three independent coders (LAJSS and HT or EGJ). Because the studies assessed smoking outcomes using dichotomous (eg, abstinence) and continuous (eg, quantity of cigarettes smoked per week or month) variables, we used two ES indices to represent the outcomes. For dichotomous outcomes, we estimated a summary odds ratio from 2 × 2 tables by calculating the odds ratio and transforming it to a log OR (with the corresponding standard error) [41]. For continuous outcomes, ESs were calculated using the standardized mean difference [42]. In the absence of means and standard deviations (SD), other statistical information (eg, *F* test) was used [33,43]. ESs (Cohen’s d) were corrected for sample size bias [44].

Multiple ESs were calculated from individual studies when the study included multiple intervention conditions (2 studies), measured more than one outcome (18 studies), used (or provided sufficient data to calculate) more than one statistical method to analyze the outcomes (ie, intent-to-treat and complete-case analysis; 19 studies), or assessed outcomes at multiple follow-ups (5 studies). ESs calculated for each intervention were treated as separate studies to avoid violating the assumption of independence [33,43]. Positive ESs indicated that participants who received the text messaging intervention reported higher smoking abstinence rates, more quit attempts, smoked fewer cigarettes per day or week, and scored lower on measures of nicotine dependence compared with a control or comparison condition. All ESs were reviewed for accuracy; discrepancies were resolved through discussion and final calculations.

### Statistical Analyses

ESs were analyzed separately by smoking outcome and stratified by type of analysis (ie, intent-to-treat or complete case analysis). The overall ES for smoking abstinence using standard and cumulative meta-analytic approaches was also assessed (a cumulative meta-analysis provides an updated pooled ES estimate each time a new trial is added). Because smoking abstinence was measured using a number of methods (point prevalence, continuous abstinence, prolonged or sustained abstinence, and repeated point prevalence), only a single measure from each study was included to avoid violating the assumption of independence [43]. Decisions regarding which ES to include for each intervention were based on the most commonly reported measure (ie, 7-day point prevalence), followed by the next common measure, and so forth until all studies reporting an abstinence measure were represented. Two studies (k=3) did not include measures of smoking abstinence [45,46].

Summary ESs were calculated using random-effects procedures such that ES were weighted by the inverse of their random-effects variance [33,47]. The homogeneity statistic, Q, was calculated; a significant Q indicates a lack of homogeneity and an inference of heterogeneity. The *I* ^2^ index and the corresponding 95% CIs were also calculated to assess the observed dispersion [48-50] The *I* ^2^ index ranges from 0% to 100% with 25%, 50%, and 75%, considered low, moderate, and high levels, respectively, of observed variance reflecting true differences in ESs [51]. Analyses were conducted in Comprehensive Meta-analysis [52] and Stata [53] using publicly available macros [33].

### Moderator Analyses

Moderator analyses examined the overall effects of smoking abstinence using a modified weighted least squares regression analyses or the meta-analytic analogue of an analysis of the variance (ANOVA) with weights equivalent to the inverse of the variance plus the random variance component for each ES [33,54,55]. Random-effects models (methods of moment) were estimated. Analyses examined a priori determined moderators of smoking abstinence. Sample and methodological characteristics (eg, gender, age, region, recruitment method), intervention type (text only vs text plus other intervention components), intervention dose and delivery (eg, number of texts sent, frequency of texts), message type (targeted), and intervention content (eg, personalized feedback, goal-setting) were examined. All moderator analyses were conducted in Comprehensive Meta-analysis [52].

### Publication Bias

Asymmetries in the distributions of ESs, indicating a possible reporting bias [56], were examined by inspecting funnel plots and assessing the degree of asymmetry [57-59]. As recommended by Stern and colleagues [60], we conducted tests for publication bias only if the dependent variable included 10 or more studies. Trim and fill procedures [61,62] are used to estimate and correct for the possibility of missing studies (based on a rank-based data augmentation procedure) if publication bias is detected using funnel plot asymmetry tests [58,59].

## Results

### Study Selection

**Figure 1:**
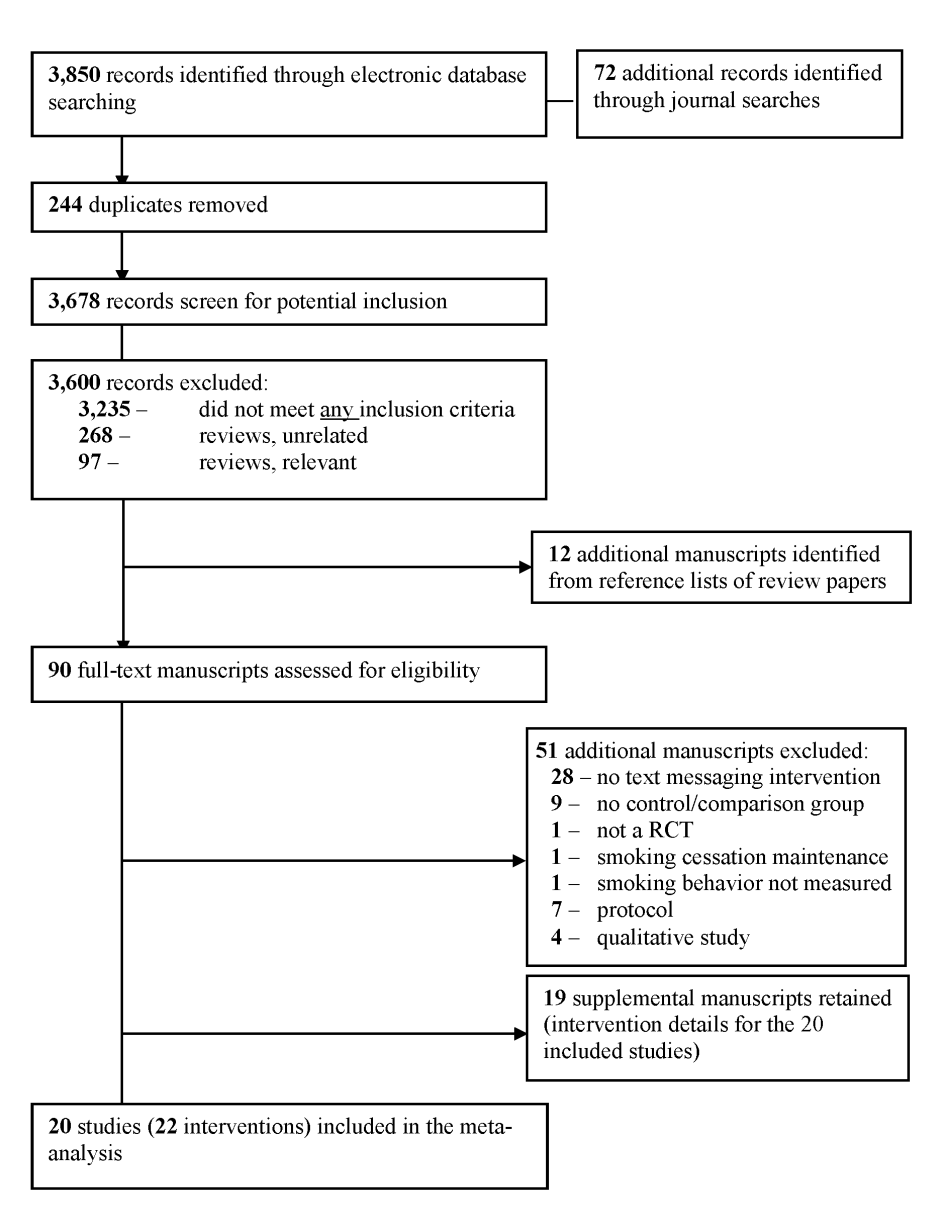
Study retrieval and selection.

Our search strategy yielded a total of 3678 unique records. Following title and abstract screening, 90 full-text manuscripts were assessed for eligibility. Of these 90 manuscript, 51 were excluded because they did not include a text messaging intervention and/or a control or comparison condition, used a quasiexperimental design, assessed only the maintenance of smoking abstinence (rather than initial smoking cessation), did not measure smoking behavior, or did not provide data suitable for meta-analysis (eg, protocol, qualitative). Our final sample included 20 studies reporting 22 interventions (k) (Figure 1). An additional 19 manuscripts were retained as supplemental information for the included studies (details of the 20 studies included in the meta-analysis are provided in Table 1).

### Study Details

The characteristics of the studies, samples, and interventions included in the meta-analysis are described below. Full details are provided in Table 2.

**Table 1:** The study, sample, and intervention characteristics for the 20 studies (22 interventions) included in the meta-analysis

Citation	Sample	Location & Recruitment	Control	Intervention	Delivery	Texts^a^	Frequency	Flow^b^	Outcomes
Abroms [63]	N^c^=503; 66% F^d^; 79% W^e^; 36 years^f^	USA; internet search engine ads	RCNM^g^	Text2Quit	Text+^h^	45	Decreasing	2	7-day PP^i^ 30-day PP^h^ repeat PP^j^
Bock [64]; Linked Studies [65]	N=60; 58% F; 65% W; 31 years	Providence, RI; community	ICM^k^	Txt2Quit	Text+	154	Varied	2	24-hour PP 7-day PP ND^l^
Borland [32]^m^; Linked Studies [66]	N=1963; 60% F; 42 years	Australia; quitline contacts, internet ads, and cold-contacts from marketing and social research data company	Info	Quit on Q	Text^n^	193	Varied; user-selected	2	7-day PP prolonged abstinence
Borland [32]^m^; Linked Studies [66]	N=1963; 60% F; 42 years	Australia; quitline contacts, internet ads, and cold-contacts from marketing and social research data company	Info	Quit on Q & QuitCoach	Text+	193	Varied; user-selected	2	7-day PP prolonged abstinence
Brendryen and Kraft [27]; Linked Studies [67,68]	N=396; 50% F; 36 years	Norway; internet newspaper ads	RCNM	Happy Ending	Text+	189	Varied	2	7-day PP repeat PP
Brendryen [26]; Linked Studies[27]	N=290; 50% F; 40 years	Norway; internet newspaper ads	RCNM	Happy Ending	Text+	189	Varied	2	7-day PP repeat PP
Buller [69]; Linked Studies [32]	N=102; 51% F; 74% W; 25 years	USA; internet social media and search engine ads	RCNM	OnQ	Text	108	Varied; user-selected	2	24-hour PP 30-day PP continuous abstinence quit attempt
Free [30]; Linked Studies [11,70-73]	N=5792; 45% F; 89% W; 37 years	United Kingdom; internet and community ads	ICNM^o^	Txt2stop	Text	225	Decreasing	2	7-day PP 28-day PP continuous abstinence^j^
Free [28]	N=200; 37% F; 36 years	United Kingdom; community ads	ICNM^o^	Txt2stop	Text	225	Decreasing	2	7-day PP^j^ 28-day PP^j^
Haug [74]	N=174; 57% F; 25 years	Germany; university	AO^p^	SMS-Coach (one weekly SMS feedback)	Text	14	Low (≤1/week, fixed-dose)	2	Quit attempt CPD^q^
Haug [74]	N=174; 57% F; 25 years	Germany; university	AO	SMS-Coach (three weekly SMS feedback)	Text	42	High (>1/week, fixed-dose)	2	Quit attempt CPD
Haug [45]; Linked Studies [75-78]	N=755; 52% F; 18 years	Switzerland; vocational schools	AO	SMS-Coach	Text	68	High (>1/week, fixed-dose); Decreasing	2	7-day PP 4-week PP CPD
Mason [46]	N=72; 43% F; 91% B^r^; 16 years	Richmond, VA; respondent driven sampling starting from a substance abuse clinic	ICM	NR^s^	Text	30	High (>1×/week, fixed-dose)	2	CPD
Naughton [79]; Linked Studies [80]	N=602; 53% F; 98% W; 42 years	England; clinics	RCNM	iQuit	Text+	108	Varied	2	continuous abstinence^j^ prolonged abstinence
Naughton [81]	N=198; 100% F; 100% W; 27 years	England; clinics	RCNM	MiQuit	Text+	80	Decreasing; varied	2	7-day PP^j^ 28-day PP quit attempt
Pollak [82]	N=31; 100% F; 49% W; 28 years	USA; clinic	RCNM	NR	Text	280	High (>1×/week, fixed-dose)	2	7-day PP^j^ CPD
Rodgers [29]; Linked Studies [83]	N=1705; 59% F; 25 years	New Zealand; internet and community ads	ICNM	NR	Text	238	Decreasing	2	7-day PP continuous abstinence CPD ND
Shi [84]	N=179; 5% F; 17 years	China; vocational schools	RCNM	NR	Text+	217	Varied	2	7-day PP 30-day PP CPD ND
Skov-Ettrup [85]; Linked Studies [86]	N=2,030; 59% F; 19 years	Denmark; newly registered users of smoking cessation website	RCM^t^	NR	Text+	80	Varied	1	30-day PP
Whittaker [31]; Linked Studies [87]	N=226; 47% F; 27 years	New Zealand; internet and community ads	ICNM	STUB IT	Text+	136	Varied	2	7-day PP continuous abstinence^j^ quit attempt
Ybarra [88]; Linked Studies [89,90]	N=151; 39% F; 36 years	Ankara, Turkey; community ads and in-person outreach in local malls	RCNM	SMS-Turkey	Text	119	Varied	1	7-day PP 30-day PP continuous abstinence^j^ CPD
Ybarra [91]; Linked Studies [88,92]	N=164; 44% F; 65% W; 22 years	USA; internet ads	RCM	SMS-USA	Text+	150	Varied	2	7-day PP continuous abstinence^j^ CPD

^a^Estimated maximum number of texts a participant could receive.

^b^One-way (1) or two-way (2) text messaging.

^c^Number of participants who began the study.

^d^Proportion female.

^e^Proportion White.

^f^Mean age in years.

^g^Relevant content, not time-matched.

^h^Text messaging plus other electronic delivery formats (eg, emails).

^i^Point prevalence.

^j^Biochemical or collateral verification of abstinence.

^k^Irrelevant content, time-matched.

^l^Nicotine dependence;

^m^The QuitCoach (n=809) and Choice (n=758) arms were excluded because participants did not (or may not have) received smoking-related text messages.

^n^Text messaging alone.

^o^Irrelevant content, not time-matched.

^p^Assessment only control.

^q^Cigarettes per day/week.

^r^Proportion Black

^s^Not reported.

^t^Relevant content, time-matched.

**Table 2:** Description of study, sample, and intervention characteristics of 20 included studies.

Characteristic				Variable	Summary Statistic
**Study**					
				Publication year, median (range)	2013 (2005-2015)
				Data collection year, median (range)	2009 (2004-2013)
**Sample**					
			Demographics		
				Sample size, initial/final	15,593/12,477
				Women, mean % (SD^a^)	54 (20)
				Age, mean (SD)	29 (8)
				Race, mean % White (SD), k^b^=9	69 (29)
			Region of sample, k (%)		
				Europe	9 (45)
				North America	6 (30)
				Oceania	3 (15)
				Asia	2 (10)
**Methods**					
			Recruitment method, k		
				Web-based	6 (30%)
				Offline	8 (40%)
				Web-based and offline	6 (30%)
			Enrollment Procedures, k (%)		
				Electronic	6 (30%)
				Phone	1 (5%)
				In-person	5 (25%)
				Multiple	8 (40%)
			Study design, k (%)		
				Random assignment of groups	2 (10)
				Matching then random assignment	8 (40)
				True randomization	10 (50)
				Treatment standardized	20 (100)
			Pre- and post-test, k (%)		
				Pretest post-test design	20 (100)
			Follow-up rate, k (%)		
				85%-100% completed	9 (45)
				70%-84% completed	6 (30)
				<70% completed	5 (25)
			Follow-up length, k (%)		
				6 months or longer	3 (15)
				3-5 months	3 (15)
				Less than 3 months	14 (70)
			Retention, k (%)		
				Withdrawal/drop-outs reported	20 (100)
				Attrition, cases lost to follow-up considered	20 (100)
			Data collection, k (%)		
				Anonymous	1 (5)
				Collateral verification	1 (5)
				Used objective measures (≥50% cases)	7 (35)
				Independent/double-blinding	11 (55)
			Data treatment, k (%)		
				Intent-to-treat, reported and used	20 (100)
			Data analyses, k (%)		
				Appropriate for the study design	2 (10)
				Controlled for baseline/other covariates	18 (90)
			Single versus multiple site study design, k (%)		
				Multisite, replication at ≥2 sites	0 (0)
**Intervention**					
				Theory used to guide research, k (%)	19 (86)
				Intervention duration (days), median (range)	87 (5-378)
			Intervention delivery, k (%)		
				Text messages	11 (50)
				Text messages + other delivery format	11 (50)
				Text messages sent, median (range)	140 (14-280)
			Frequency of text messages, k^c^		
				Low (<1/week), fixed-dose	1
				High (>1/week), fixed-dose	4
				Decreasing	6
				Varied	13
				User selected	3
	Communication flow, k (%)				
				One-way texts	2 (9)
				Two-way texts	20 (91)
		Other Intervention Content (k=11)			
				N sessions, median (range)	2 (1-232)
		Other Intervention Delivery, k^c^			
				In-person	2
				Facilitated by computer/technology	1
				Computer/technology	6
				Print materials	2
				Phone	4
		Tailored and Targeted Intervention			
				Intervention content tailored	21 (95)
				Intervention content targeted	16 (73)
		Other Intervention Content, k (%)			
				Decisional balance exercise	12 (55)
				Personalized feedback	16 (73)
				Self-efficacy	20 (91)
				Self-management skills	22 (100)
				Goal-setting/harm prevention plans	19 (86)
		Counseling provided, k (%)^c^			
				In-person	1 (5)
				Phone/voice	3 (14)
				Computer	3 (14)
		Social support, k (%)			
				Any	12 (55)
				Individual	11
				Group	1
		Biomedical intervention, k (%)			
				Any	9 (41)
				Recommended	7
				Provided	2
		Treatment fidelity, k (%)			
					15 (68)
**Controls**					
	Type of control, k (%)				
				WL/NT/AO^d^	2 (10)
				Irrelevant content, time-matched	2 (10)
				Irrelevant content, not time-matched	4 (20)
				Relevant content, time-matched	2 (10)
				Relevant content, not time-matched	10 (50)
		Control delivery, k (%)			
				Text messages	6 (33)
				Text messages + other delivery format	4 (22)
				Other delivery format	8 (44)

^a^Standard deviation.

^b^Number of studies.

^c^Multiple categories were possible.

^d^Wait-list/no treatment/assessment only control.

#### Study and Sample Characteristics

Studies were published (or indexed as an advance online publication) in journals between 2005 and 2015 (median publication year=2013) with data collection occurring an average of 3 years earlier (median data collection year=2009; range, 2004-2013). (All studies available through December 31, 2014 were included in the meta-analysis but one study indexed as an advance online publication was subsequently published during the preparation of this manuscript [46].) Participants were recruited using multiple methods including web-based (eg, Internet advertisements, quitlines (6/20, 30%)), offline (eg, clinics, schools or universities; (8/20, 40%)), or a combination of web-based and offline approaches (eg, internet and community advertisements; (6/20, 30%)). The study samples were located in 10 countries: United States (6), United Kingdom (4), Germany (1), New Zealand (2), Norway (2), Turkey (1), Australia (1), China (1), Denmark (1), and Switzerland (1). Of the 15,593 smokers who consented to participate in the studies, more than one-half were women (8128/15,593, 54%), most were White (mean=69%, SD=0.29), and averaged 29 years of age (range=16-42). The mean retention rate was 78% (SD=0.17).

#### Control Conditions

The interventions were most often compared with an active comparison condition (18/20, 90%). Many of the active comparison conditions included smoking-related content (12/18, 67%) but were infrequently matched for time and contact (4/20, 22%). The active comparison conditions included content not delivered via text messaging (8/20, 44%), text messaging (6/20, 33%), and text messaging plus other components (4/20, 22%).

#### Text Messaging Interventions

Most text messaging interventions were guided by theory (19/22, 86%). Of the 22 interventions evaluated, 59% (13/22) reported using more than one theory to guide the intervention development. A wide range of theories were reported but most often included the Transtheoretical model [93] (11/22, 50%), Social Cognitive Theory [94] (10/22, 45%), and cognitive behavioral therapy [95,96] (5/22, 23%).

Interventions were delivered over a median of 87 days (range, 5-378 days) and included texts messages alone (11/22, 50%) or text messaging plus intervention content delivered using another modality (13/22, 50%). The maximum number of texts messages that a smoker could receive averaged 140 (SD=76) across studies and ranged from 14 to 280 messages. The frequency of these text messages was most often varied (13/22, 59%) and two-way communication (20/22, 91%) was typically allowed. Treatment fidelity (ie, receipt of text messages) was assessed in 68% (15/22) of the studies. Of the 11 interventions that supplemented the text messages with other intervention content, most were delivered entirely via computer/Internet (eg, online chat rooms, smoking-related modules available via the study website; 6/11; 36%) and included a median of two sessions (range, 1-232; k=7).

Most text messaging interventions were targeted (16/22, 73%) to the sample (eg, pregnant smokers) and tailored (21/22, 95%) to the recipient (eg, quit date set by the individual smoker). The interventions often provided personalized feedback on smoking behaviors (16/22, 73%), encouraged participants to set quit goals or make plans to reduce smoking (19/22, 86%), addressed self-efficacy to reach smoking cessation goals (20/22, 91%), and provided self-management skills training (22/22, 100%). Participants were often encouraged to use social support (12/22, 55%) and 41% (9/22) provided or recommended pharmacological interventions (eg, nicotine patch) to aid in their smoking cessation.

#### Methodological Quality

Studies satisfied an average of 65% (SD=0.09) of the methodological quality criteria, indicating moderate methodological quality. All studies used a RCT, standardized the intervention content (ie, using an intervention manual, facilitator training), and measured smoking behaviors at baseline. Most studies included a follow-up assessment that was administered less than 3 months post-intervention (14/20, 70%) and retained at least 85% (17/20) of the study participants at the final post-intervention assessment (9/20, 45%). Studies rarely obtained collateral verification (1/20, 5%) or used objective measures in at least one-half of the sample (eg, verification of smoking behaviors by testing levels of cotinine or carbon monoxide; 7/20, 35%) to validate self-report measures of smoking cessation. Because most studies personalized text-messages participants received, anonymity could not be ensured (19/20, 95%). Many studies (11/20, 55%) used study personnel who were blind to the group assignment. Studies reported participant flow (ie, withdrawals and attrition; 20/20, 100%). Statistical analyses often controlled for baseline or other characteristics (18/20, 90%) and all studies (20/20, 100%) used an intent-to-treat approach. The proportion of methodological quality that satisfied the criteria was not associated with overall smoking abstinence, B=0.44 (SE=0.45), *P*=.327, Q_R_=0.07, *P*=.789.

### Efficacy of Text Messaging Interventions by Smoking Outcomes

#### Overview of the Results

Because only five studies assessed smoking cessation at multiple post-intervention assessments [32,46,64,69,91], we used the last post-intervention assessment from each study in the analyses. All studies included post-intervention assessments of smoking cessation including 18 studies (k=19) that measured abstinence (2: 24-hour point prevalence; 14 (k=15): 7-day point prevalence; 9: 4-week point prevalence; 7 continuous abstinence; 2 (k=3) prolonged or sustained abstinence; 3: repeated point prevalence abstinence), 4 studies (k=5) measured quit attempts any time during the intervention period, 8 studies (k=9) assessed cigarette consumption, and 3 studies assessed nicotine dependence. (The summary ESs and homogeneity analyses by smoking cessation measures and type of analysis are presented in Table 3).

**Table 3:** Summary effect sizes and homogeneity statistics (random effects assumptions) at the final post-intervention assessment for smoking abstinence and quit attempts.

Analyses	Outcome	k^a^	OR^b^ (95% CI^c^)	Q^d^	*P* Value	*I* ^2e^ (95% CI)
Intent-to-Treat						
	Point prevalence, 24 hours	2	2.60 (1.26, 5.37)	0.14	.704	0 (0, 100)
	Point prevalence, 7 days	16	1.38 (1.22, 1.55)	18.34	.245	18 (0, 55)
	Point prevalence, 30 days	9	1.52 (1.34, 1.71)	5.72	.678	0 (0, 63)
	Continuous abstinence	7	1.63 (1.19, 2.24)	11.31	.079	47 (0, 78)
	Prolonged abstinence	3	1.57 (1.19, 2.08)	0.80	.671	0 (0, 89)
	Repeated point prevalence	3	2.33 (1.61, 3.38)	1.52	.467	0 (0, 58)
	Quit attempt	5	1.15 (0.84, 1.57)	4.35	.360	8 (0, 47)
Complete case						
	Point prevalence, 24 hours	2	3.62 (1.46, 8.99)	0.02	.895	0 (0, 100)
	Point prevalence, 7 days	15	1.43 (1.31, 1.56)	13.55	.484	0 (0, 0)
	Point prevalence, 30 days	9	1.57 (1.39, 1.77)	7.47	.487	0 (0, 100)
	Continuous abstinence	7	1.92 (1.55, 2.38)	6.88	.332	13 (0, 56)
	Prolonged abstinence	3	1.57 (1.19, 2.07)	0.45	.798	0 (0, 95)
	Repeated point prevalence	3	2.33 (1.60, 3.39)	1.41	.495	0 (0, 57)
	Quit attempt	5	1.33 (0.83, 2.13)	4.72	.317	15 (0, 60)

^a^Number of interventions

^b^Odds ratios; greater than 1 indicate that the estimated effects favor the text messaging interventions relative to controls.

^c^confidence interval.

^d^Homogeneity statistic.

^e^Consistency of effect sizes.

#### Smoking Abstinence

Smokers who received a text messaging intervention were more likely to abstain from smoking relative to controls across a number of smoking abstinence measures (point prevalence, continuous abstinence, prolonged abstinence, and repeated point prevalence). The magnitudes of the summary ESs were larger when complete case analyses were used (see Table 3).

#### Quit Attempts

Quit attempts were measured at the post-intervention in five studies. There were no differences in quit attempts between text messaging and control groups using intent-to-treat or complete case analyses.

#### Cigarette Consumption

The number of cigarettes smoked per day or week was measured in eight studies (k=9; 6 complete case, 3 intent-to-treat). Smokers who received a text messaging intervention reported smoking fewer cigarettes per day or week compared with controls, d_+random_=0.17, 95% CI=0.07, 0.28 (complete case analysis). The hypothesis of homogeneity was supported: Q_5_=5.89, *P*=.317, *I* ^2^=15 (0, 60)*.* There were no significant differences between the intervention and control groups using an intent-to-treat approach (d_+random_=0.07, 95% CI=−0.17, 0.31, Q_2_=2.28, *P*=.320, *I* ^2^=12, 95% CI=0, 53). The overall analyses (k=9) indicated that participants reported smoking significantly fewer cigarettes per day or week if they received a text messaging intervention versus a control condition: d_+random_=0.14, 95% CI=0.05, 0.23. The hypothesis of homogeneity was supported for cigarette consumption: Q_8_=8.60, *P*=.377; *I* ^2^=7 (95% CI=0, 45).

#### Nicotine Dependence

Nicotine dependence was assessed in three studies using a complete case analysis (none of the studies supplied enough information for intent-to-treat analyses for nicotine dependence). There were no significant differences between the intervention and control groups on nicotine dependence at the post-intervention assessment: d_+random_=0.00, 95% CI=−0.39, 0.39, Q_2_=7.67, *P*=.022, *I* ^2^=74, 95% CI=13, 92).

### Standard and Cumulative Analyses of Smoking Abstinence

**Figure 2:**
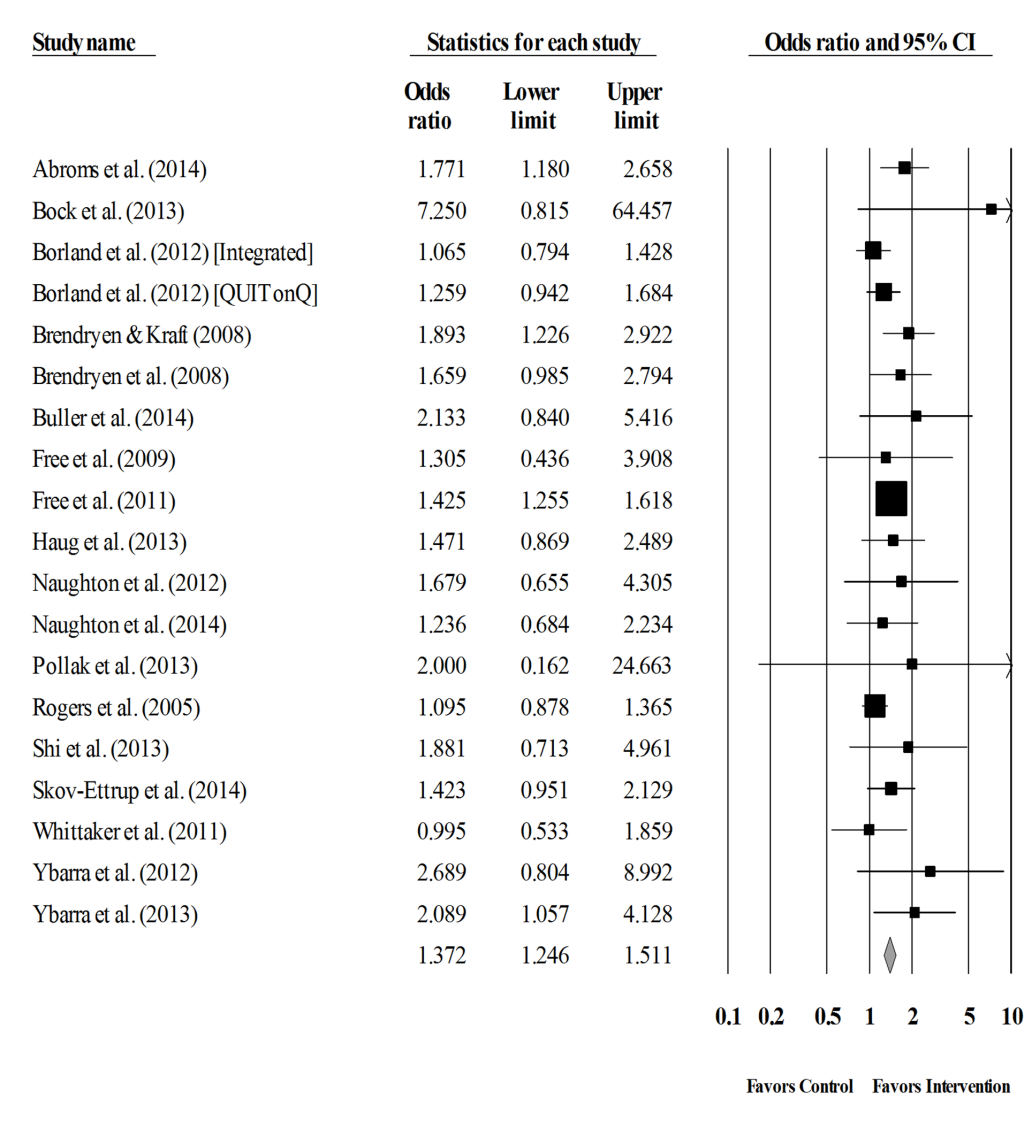
Forest plot of the overall odds ratio and the corresponding 95% confidence intervals for smoking abstinence. The size of the square representing the odds ratio for each study is proportional to its weight in the analysis.

The overall summary ES for smoking abstinence was significant, OR=1.37 (95% CI=1.25, 1.51; k=19). That is, participants who received a text messaging intervention were 1.37 times more likely to abstain from smoking relative to controls. The hypothesis of homogeneity was supported: Q_18_=19.36, *P*=.370. *I* ^2^ was 7% (95% CI 0, 42). The confidence intervals surrounding *I* ^2^ did not exceed the 50% threshold indicating that the proportion of observed heterogeneity is low (a forest plot of the overall smoking abstinence is provided in Figure 2).

**Figure 3:**
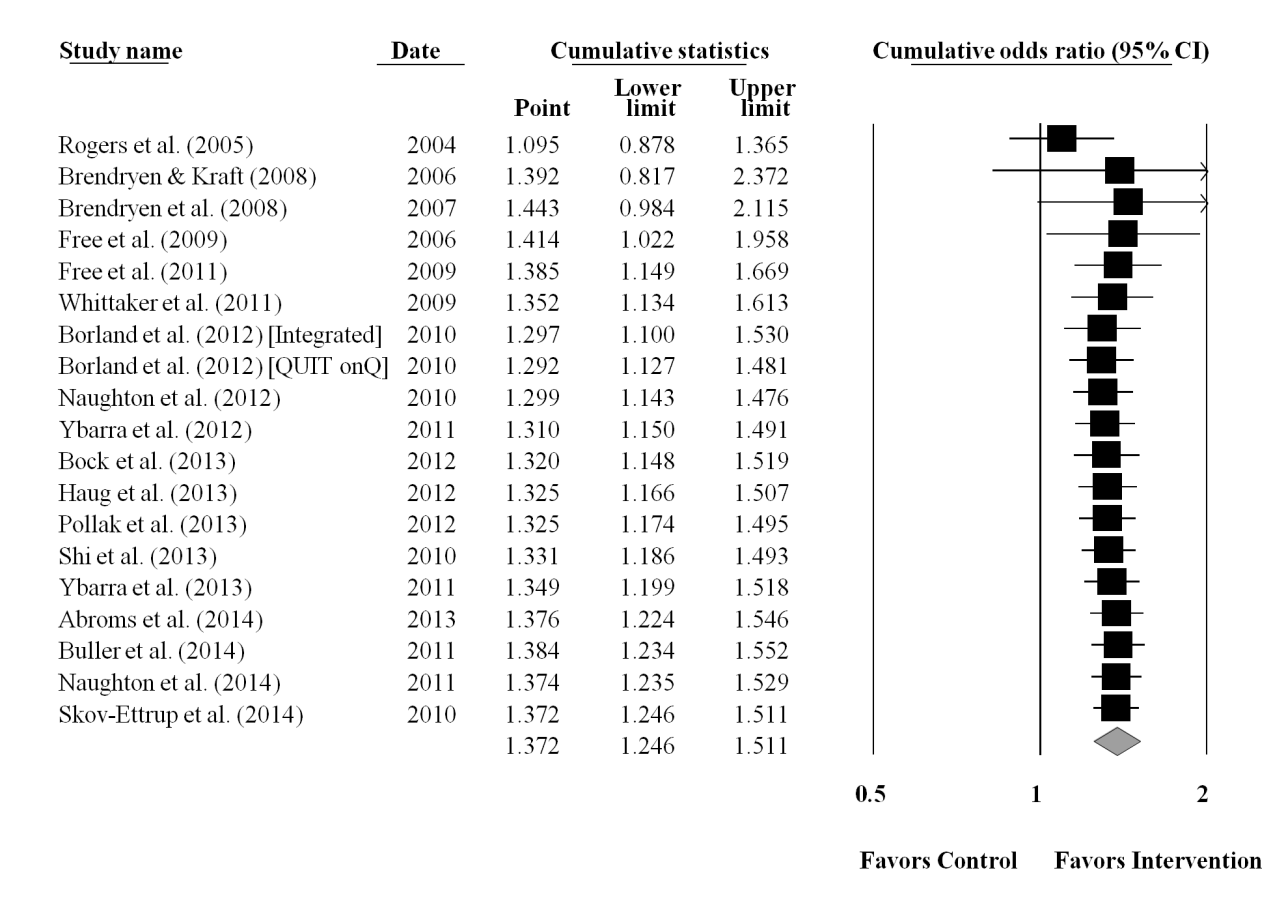
Cumulative plot of the overall weighted mean effect sizes and the corresponding 95% confidence intervals for smoking abstinence, based on final date of data collection.

**Figure 4:**
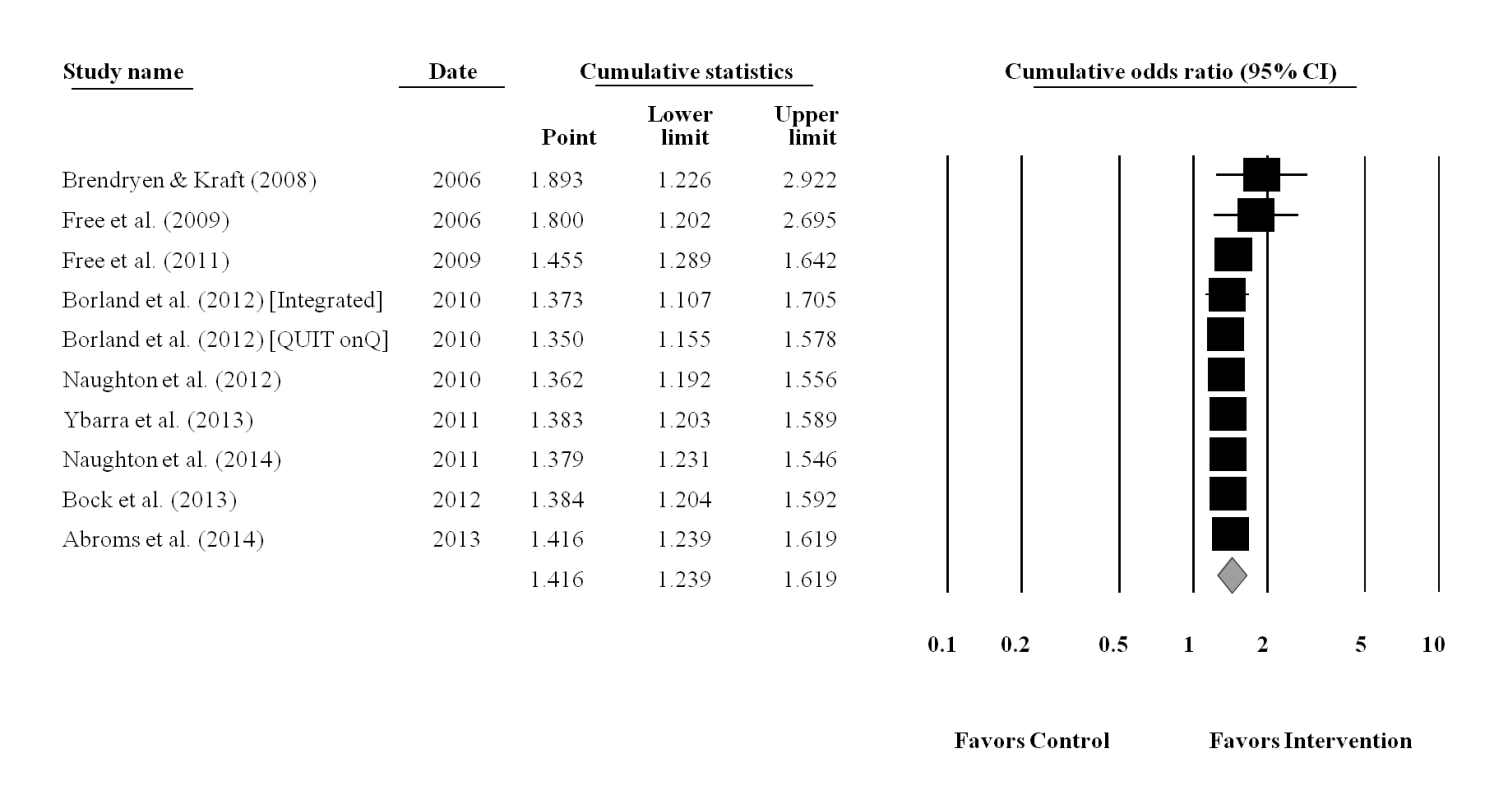
Cumulative plot of the overall weighted mean effect sizes and the corresponding 95% confidence intervals for smoking abstinence, based on final date of data collection and restricted to studies with moderate to high methodological quality ratings.

The cumulative meta-analysis was performed using the final completion date for data collection for each study (Figure 3). Results showed that the benefits of using a text messaging approach for smoking cessation was established by 2009 (end of data collection for Free et al [30]), after only five RCTs involving 8383 smokers (OR=1.39, 95% CI=1.15, 1.67, *P*<.001). Results from the additional 13 studies (k=14) with 6870 participants did not change the established efficacy of text messaging interventions for smoking cessation. The CIs surrounding the ES estimates narrowed as the data accumulated. Exploratory analyses restricted to the 10 studies that were of moderate to high methodological quality (ie, studies satisfying at least 65% of the methodological quality criteria; median=70%, range, 65%-80%) also indicated that the efficacy of text messaging for smoking cessation was established by 2009 (end of data collection for Free et al [30]), after only three moderate to high quality studies involving 6388 smokers, OR=1.46, 95% CI=1.29, 1.64. A cumulative plot for smoking abstinence restricted to studies meeting criteria for moderate to high methodology quality is provided (Figure 4).

**Table 4:** Moderators of overall smoking abstinence at the final available assessment.^a^

Characteristics		Moderators	k^b^	B (SE)	OR^c^ (95% CI^d^)	Q_B_^e^
**Sample**						
		Women, %	19	0.58 (0.26)*		1.07
		Mean Age	19	0.39 (0.24)		0.10
	Region of Sample					14.38**
		Europe	8		1.46 (1.31, 1.62)	
		North America	5		1.94 (1.41, 2.67)	
		Oceania	4		1.12 (0.97, 1.30)	
		Asia	2		2.16 (1.02, 4.61)	
**Methods**						
		Methodological quality rating	19	0.44 (0.45)		0.07
	Recruitment					6.39*
		Web-based	6		1.72 (1.41, 2.11)	
		Offline	6		1.45 (1.05, 2.00)	
		Web-based and offline	7		1.30 (1.18, 1.43)	
**Intervention**						
		Intervention duration, no. days	19	0.21 (0.09)*		1.48
	Intervention type					0.67
		Text	8		1.33 (1.17, 1.52)	
		Text+	11		1.45 (1.23, 1.70)	
		Text messages, n sent	19	0.54 (0.16)***		2.24
	Frequency of texts					0.10
		Varied	13		1.40 (1.20, 1.64)	
		Other	6		1.36 (1.17, 1.57)	
	Communication flow					0.27
		One-way	2		1.53 (1.02, 2.28)	
		Two-way	17		1.37 (1.23, 1.52)	
	Intervention targeted					2.06
		Yes	15		1.41 (1.28, 1.55)	
		No	4		1.20 (0.98, 1.47)	
	Provided counseling					3.41
		Yes	3		1.85 (1.33, 2.58)	
		No	16		1.34 (1.23, 1.46)	
	Decisional balance exercise					0.09
		Yes	9		1.41 (1.18, 1.69)	
		No	10		1.36 (1.20, 1.55)	
	Personalized feedback					0.00
		Yes	13		1.38 (1.23, 1.54)	
		No	6		1.39 (1.07, 1.80)	
	Self-efficacy addressed					0.09
		Yes	17		1.37 (1.24, 1.52)	
		No	2		1.49 (0.88, 2.54)	
	Social support					0.89
		Yes	11		1.42 (1.26, 1.59)	
		No	8		1.28 (1.07, 1.53)	
	Biomedical intervention					0.71
		Yes	9		1.42 (1.25, 1.60)	
		No	10		1.30 (1.11, 1.53)	
	Active control					2.37
		Yes	16		1.42 (1.29, 1.55)	
		No	3		1.20 (0.99, 1.45)	

^a^Meta-regression (continuous variables) and the meta-analytic analogue to the ANOVA (categorical variables) homogeneity analysis were conducted to examine potential moderators of smoking abstinence. All moderator tests are based on random-effects models.

^b^Number of interventions.

^c^Summary odds ratio.

^d^confidence interval.

^e^Homogeneity test for between-groups.

*P<.05; **P<.01; ***P<.001.

### Moderators of Smoking Abstinence

Moderator tests were conducted for overall smoking abstinence (Table 4). Compared with controls, text messaging interventions were more successful in increasing smoking abstinence when the trials included fewer women (B=0.58, SE=0.26, *P*=.025) and were conducted in Asia (OR=2.16, 95% CI=1.02, 4.61), North America (OR=1.94, 95% CI=1.41, 2.67), or Europe (OR=1.46, 95% CI=1.31, 1.62) versus Oceania (OR=1.12, 95% CI=0.97, 1.30), Q_3_=14.38, *P*=.002. Text messaging interventions were more successful at increasing smoking abstinence when the participants were recruited via the Internet (eg, Web-based advertisements, quitline; OR=1.72, 95% CI=1.41, 2.11) versus offline (eg, schools or clinics; OR=1.45, 95% CI=1.05, 2.00) or via a combination of Web-based and offline approaches (eg, Internet and community ads; OR=1.30, 95% CI=1.18, 1.43), Q_2_=6.39, *P*=.041.

### Risk of Publication Bias

Both graphical and statistical tools were used to test for the possibility of publication bias. Results from Begg’s test [58] and Egger’s regression asymmetry test [59] revealed no evidence of publication bias for the dependent variables with sufficient cases for assessment (ie, 7-day point prevalence abstinence and overall smoking abstinence). The funnel plots and results of the statistical tests are available in Multimedia Appendix 2.

## Discussion

### Primary Findings

This meta-analysis evaluated the impact of text messaging interventions on smoking outcomes among 20 RCTs reporting on 22 interventions among 15,593 smokers. The results of this meta-analysis provide evidence for the efficacy of text messaging interventions on smoking outcomes. The overall odds of smoking abstinence were 1.37 times higher in the text messaging versus control or comparison groups. This finding is comparable to other meta-analyses evaluating text messaging interventions for smoking cessation [23-25]. Furthermore, our cumulative meta-analysis showed that the benefits of text messaging interventions for smoking cessation were established by 2009, after only five RCTs involving 8383 smokers and culminating with Free et al [30], although the results of the trial were not published until 2011. Most of the subsequent RCTs were already underway or completed by 2011. The robustness of this finding (coupled with limited evidence of heterogeneity) clearly indicates that conducting any future RCTs with the primary goal of assessing the efficacy of text messaging for smoking cessation is unnecessary.

It is noteworthy that the summary ESs favored the treatment groups even when 90% (18/20) of the controlled trials used an active control and 67% (12/18) of these active controls included some smoking-related content. These active controls with smoking-related content used a variety of means to disseminate smoking cessation information including smoke-free websites, self-help guidebooks, and smartphone apps. Some of the active controls included in the overall smoking abstinence analyses also provided text messaging, but the text messaging was a weaker form of that offered to the intervention groups or contained unrelated content (eg, diet and physical activity, importance of study participation). For example, Pollak et al [82] compared text messaging support messages that used a scheduled, gradual smoking reduction approach with standard smoking-related text messaging support messages and Free et al [30] compared smoking-related text messages with study-related participation texts (eg, thanking participants for taking part in the study, requests for updating contact details). Further probing revealed that the use of an active comparison condition with or without text messaging did not moderate smoking abstinence (active comparisons with any text messages: OR=1.37, 95% CI=1.17, 1.61, k=8; active comparisons without text messages: OR=1.39, 95% CI=1.18, 1.64, k=10; Q_1_=0.02, *P*=.897). Furthermore, there was no difference between active comparisons that included smoking-related (OR=1.55, 95% CI=1.02, 2.38, k=3) or nonsmoking (OR=1.35, 95% CI=1.10, 1.66, k=5) text messaging, Q_1_=0.38, *P*=.561. Thus, our meta-analysis provides evidence that text messaging interventions for smoking cessation improves smoking abstinence above and beyond other (weaker) smoking cessation delivery modalities with or without text messaging.

Our meta-analysis also demonstrated that the magnitudes of the summary ESs across measures of smoking abstinence were weaker in text messaging studies using an intent-to-treat analytic approach versus a complete case analysis (ORs ranged from 1.38-2.60 vs 1.43-3.62). Excluding participants after randomization from analyses introduces bias that may alter the conclusions made about individual studies’ treatment effects [34]. Failure to account for these biases can also affect meta-analytic results. Future studies should include intent-to-treat analyses when presenting their results to minimize potential biases, and perhaps make efforts to examine patterns of attrition.

The results from our moderator analyses revealed three important moderators of text messaging interventions for smoking cessation. First, text messaging interventions conducted in North America, Europe, and Asia produced better results than those conducted in Oceania. All of the RCTs located in North America were conducted in the United States Over the past 20 years, smoking has become far less socially acceptable in the Unites States than in previous decades [97]. Smoking is no longer permitted in government offices and other government facilities, and in most of the Unites States smoking is not permitted in restaurants and many other public venues [98]. Smoke-free laws are also in place in many European Union countries, and many countries represented in this study have completely banned smoking in public places, workplaces, or on public transportation [99]. Smoke-free environments have also been introduced in many Asian countries such as China and Turkey [100,101]. It may be that light-touch/low-intensity interventions such as text messaging are most effective when the surrounding environment supports cessation, or at least is actively unsupportive of continued smoking.

Second, the efficacy of the text messaging interventions for smoking cessation differed by men and women. That is, studies with larger proportions of women participants were less successful at improving smoking abstinence. Prior research has suggested that interventions to increase smoking cessation may be less effective for women than men [102]. Women may be less likely than men to quit smoking for a number of reasons including weight concerns, less social support for quitting, genetic variants that affect the efficacy of pharmacotherapies, and mood regulation [103]. Interventions that specifically address women’s concerns can help women stop smoking [104]. Only two text messaging interventions included in this meta-analysis were targeted to women, specifically pregnant smokers, and found mixed results [81,82]. One study found no differences in smoking abstinence between the text messaging intervention and no message control group, while the other study showed a significant difference in smoking abstinence among women who received text messages that used a scheduled gradual smoking reduction approach versus support text messages alone. Future text messaging interventions for smoking cessation should address the specific treatment needs of women with further attention to the type and intensity of the text messages desired by women smokers.

Finally, recruitment method was associated with increases in smoking abstinence. Studies in which participants were recruited exclusively via the Internet achieved higher rates of smoking abstinence relative to studies that recruited participants using an offline or combination of Web-based and offline recruitment methods. It may be that Web-based recruitment is more culturally consistent with use of a technology-delivered intervention, and individuals who respond to Web-based recruitment may be more comfortable with and better able to respond to a text-message delivered program compared with other individuals. More work is needed to identify the characteristics of individuals who do well with technology-delivered interventions versus those who would respond better to more traditional (eg, in-person) therapies.

Prior research comparing four Web-based (3: health risk assessment; advertisements; quit line screening) and offline (1: offline materials such as television advertisements) recruitment methods also showed that Web-based advertisements had higher yield rates and were more cost-effective than other approaches [105]. Despite the potential benefits of Web-based recruitment, other research shows that participants recruited via the Internet (vs offline) communication are less likely to participate through follow-up [106]. Retention rates for studies using Web-based, offline, and combined recruitment methods in this meta-analysis were 70%, 83%, and 78%, respectively. Retaining smokers in smoking cessation interventions is an ongoing concern but these concerns may be mitigated by the added convenience, potential participation rates, and cost-effectiveness of Web-based recruitment. Nonetheless, text messaging interventions for smoking cessation should employ strategies known to be effective for increasing retention (eg, emphasizing the benefits of participation, reminders [107]) when recruiting smokers via the Internet.

### Limitations

Several limitations should be considered when interpreting our findings. One limitation of evaluating the effect of smoking cessation interventions is that studies used many different measures (eg, variety of measures of abstinence such as point prevalence and continuous abstinence; for a discussion, see [39]). Because some of these measures (eg, repeated point prevalence) were used in a limited number of studies, we could not assess moderators of these individual types of measures separately and necessitated pooling measures for our overall analyses. Second, evaluation of this literature is also limited as few of these studies systematically assessed the additive effect of text messaging. That is, most of the text messaging interventions included text messaging plus some other smoking-related content (eg, counseling, supporting website) rather than testing the effects of the same smoking cessation intervention with or without text messaging. Future studies should examine the additive effects of text messaging plus other smoking-related content. Third, identification and retrieval of relevant studies may have been hindered by the author’s use of keywords (eg, failure to include ‘intervention’ as a keyword because the intervention modality was the focal point of the study). To reduce the possibility of inadvertently missing studies, we searched multiple electronic databases, tables of contents of relevant journals, and reference sections of relevant papers and reviews [108]. Fourth, we focused our analyses on the final post-intervention assessment because most of the studies (15/20, 75%) did not provide data from multiple post-intervention assessments. Using the last post-intervention assessment as the point of analysis provides a stronger test of the potential effects of the intervention on long-term cessation because initial intervention effects tend to decay over time (cf. Johnson et al [109]). In this meta-analysis, however, the final post-intervention assessment occurred most often immediately following the completion of the intervention (median=1 week; range, 0-44 weeks). Future research with follow-up periods extended to 1 year or longer is needed to determine whether reductions in smoking behavior are sustained over time. Finally, our moderator tests were limited to the available data. Potentially important moderators could not be tested because there were too few or too many cases to be evaluated (eg, 22/22, 100% of the interventions provided self-management training), and thus were omitted from our analyses.

### Conclusion

The prevalence of cigarette smoking has declined over the past decade; however, more than 1 billion adults continue to smoke worldwide [1]. Text message interventions to reduce tobacco use have the promise to reach a wider audience with minimal cost and fewer resources. The current meta-analytic review provides unequivocal support for the efficacy of text messaging interventions for smoking abstinence. Future research should be directed to understanding for whom and under what circumstances text messaging interventions are optimized, and the duration of the effects.

## Appendix

Multimedia Appendix 1 PRISMA Checklist.

Multimedia Appendix 2 Publication Bias.

## Abbreviations

ANOVA: analysis of the variance
AO: assessment only
CPD: cigarettes per day/week
CI: confidence interval
ES: effect size
ICM: irrelevant content, time-matched
ICNM: irrelevant content, not time-matched
NR: not reported
ND: nicotine dependence
OR: odds ratio
PP: point prevalence
PRISMA: Preferred Reporting Items for Systematic Reviews and Meta-Analyses
RCT: randomized control trial
RCNM: relevant content, not time-matched
RCM: relevant content, time-matched
RR: risk ratios
SD: standard deviation
SMS: short message service
Q: homogeneity statistic
