# Supplementary material for: Text Messaging-Based Interventions for Smoking Cessation: A Systematic Review and Meta-Analysis
Source: JMIR Mhealth Uhealth. 2016 May 20;4(2):e49. doi: 10.2196/mhealth.5436 (PMC4893152; doi:10.2196/mhealth.5436)
Supplement: Multimedia Appendix 2 [file mhealth_v4i2e49_app2.pdf]

Multimedia Appendix 2: Publication Bias

Figure 2A. Funnel Plot to Assess Asymmetries in Effect Size Estimates for Smoking Abstinence, 7-day point prevalence (Intent-to-Treat).

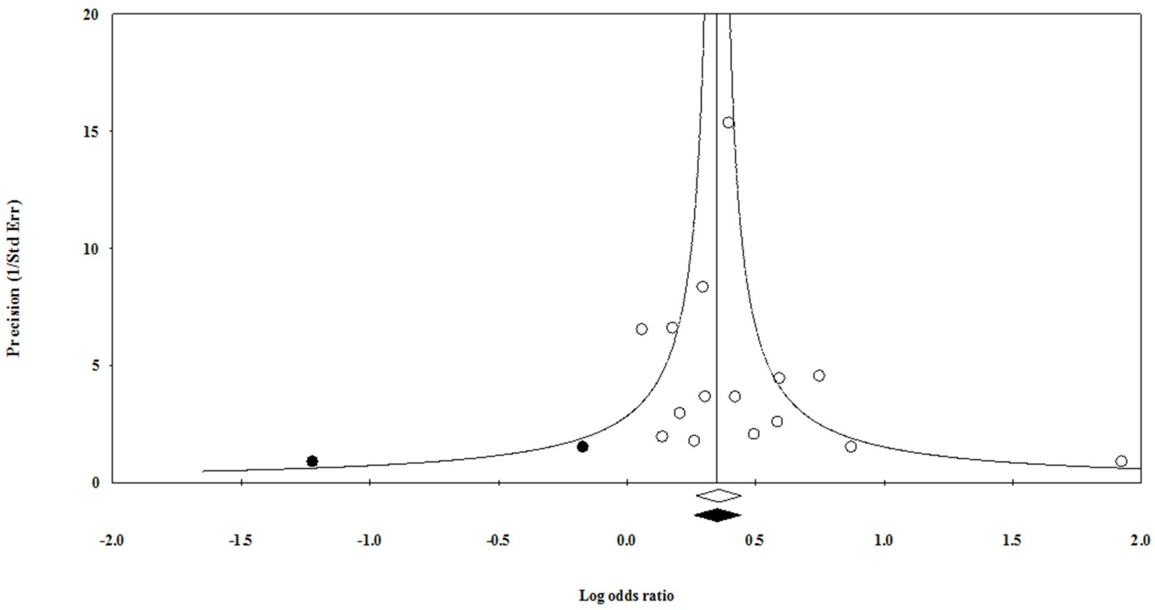

Figure 2B. Funnel Plot to Assess Asymmetries in Effect Size Estimates for Smoking Abstinence, 7-day point prevalence (Complete Case).

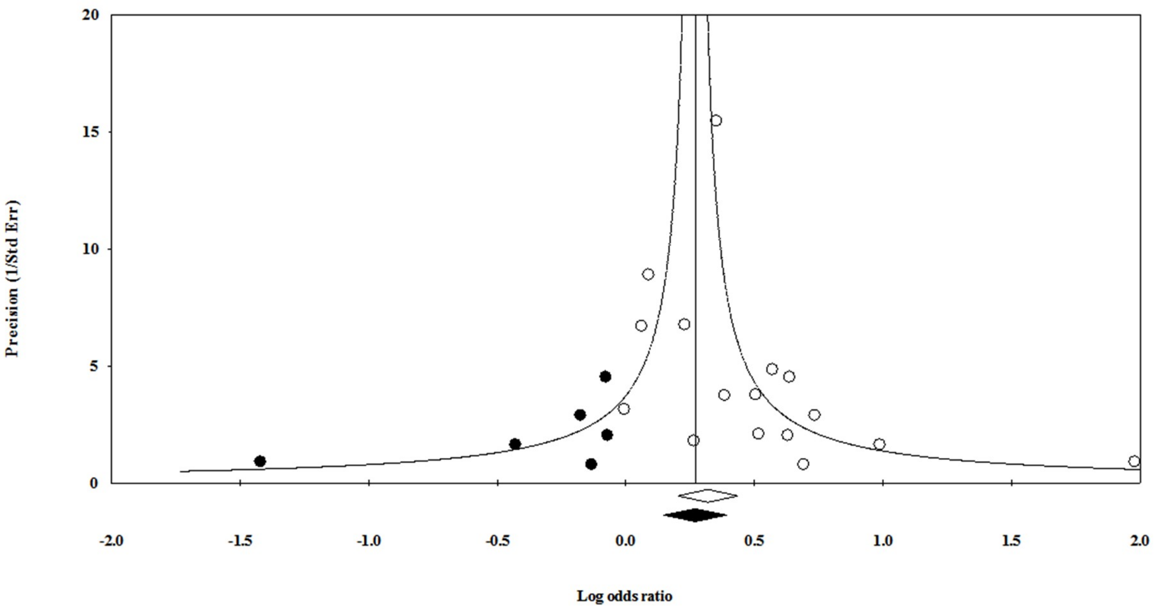

Figure 2C. Funnel Plot to Assess Asymmetries in Effect Size Estimates for Smoking Abstinence, Overall.

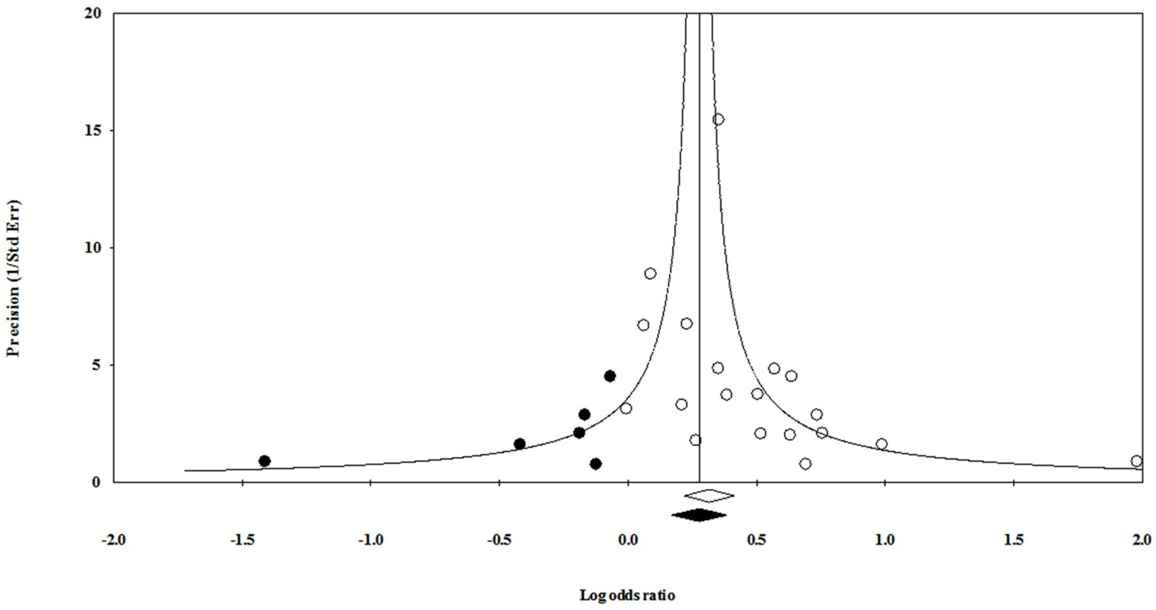

Table 2A. Regression analyses testing funnel plot asymmetry[a]

		Egger's method		Begg's method	
	k	Intercept (95% CI)	P	Kendall's tau	P
Smoking Abstinence					
Point prevalence, 7 days (ITT)	16	0.66 (-0.22, 1.55)	.130	0.16	.392
Point prevalence, 7 days (CC)	15	0.33 (-0.56, 1.22)	.439	0.19	.322
Overall Smoking Abstinence	19	0.66 (-0.10, 1.41)	.085	0.22	.184

[a]ITT, intent-to-treat. CC, complete case. k, number of interventions. CI, confidence intervals.
